# Efficacy and safety of PD-1/PD-L1 inhibitors combined with anti-angiogenic therapy for the unresectable hepatocellular carcinoma and the benefit for hepatitis B virus etiology subgroup: a systematic review and meta-analysis of randomized controlled trials

**DOI:** 10.1186/s12885-023-10960-w

**Published:** 2023-05-24

**Authors:** Danxue Huang, Liyuan Ke, Hongxia Cui, Su Li

**Affiliations:** Department of Pharmacy, Cancer Hospital of China Medical University, Liaoning Cancer Hospital and Institute, Shenyang, China

**Keywords:** PD-1 inhibitor, PD-L1 inhibitor, Anti-angiogenic, Hepatitis B Virus, Hepatocellular carcinoma, Meta-analysis

## Abstract

**Background:**

Hepatocellular carcinoma (HCC) is the third leading cause of cancer-related death, worldwide. The predominant causative factor for HCC is hepatitis B virus (HBV) infection. We conducted a meta-analysis to estimate the efficacy and safety of PD-1/PD-L1 inhibitors combined with anti-angiogenic therapy for the first-line treatment of the unresectable HCC and to evaluate the benefits of different geographic regions and etiology stratifications.

**Methods:**

Randomized clinical trials published up to 12th November 2022 were searched by online databases. Moreover, effects of hazard ratio (HR) for overall survival (OS) and progression-free survival (PFS) were extracted from included studies. Pooled odds ratio (OR) and 95% CI for objective response rate (ORR), disease control rate (DCR), and treatment-related adverse events (TRAEs) were calculated.

**Results:**

A total of 3057 patients from five phase III randomized clinical trials were collected and reviewed for this meta-analysis. The pooled HR of OS (HR = 0.71; 95% CI: 0.60–0.85) and PFS (HR = 0.64; 95% CI: 0.53–0.77) demonstrated significantly better benefit in PD-1/PD-L1 inhibitors combination group than targeted monotherapy to treat unresectable HCC. In addition, combination therapy showed better ORR and DCR, with ORs of 3.29 (95% CI: 1.92–5.62) and 1.88 (95% CI: 1.35–2.61), respectively. The subgroup analysis indicated that PD-1/PD-L1 inhibitors combination therapy was significantly superior to anti-angiogenic monotherapy for HBV-related HCC in terms of OS (HR = 0.64; 95% CI: 0.55–0.74) and PFS (HR = 0.53; 95% CI:0.47–0.59), while there was no significant difference in patients with HCV (OS, HR = 0.81, *p* = 0.1) or non-viral (OS, HR = 0.91, *p* = 0.37; PFS, HR = 0.77, *p* = 0.05).

**Conclusions:**

Meta-analysis revealed for the first-time that PD-1/PD-L1 inhibitors combination therapy for unresectable HCC was associated with better clinical outcomes than anti-angiogenic monotherapy, especially for HBV infection and Asian population.

**Supplementary Information:**

The online version contains supplementary material available at 10.1186/s12885-023-10960-w.

## Introduction

Hepatocellular carcinoma (HCC) is the major type of primary liver cancer and the third leading cause of cancer-related death worldwide [1]. Although early-stage tumor can be curable by surgical resection, ablation, or liver transplantation [2], the vast majority of patients had advanced unresectable disease at time of initial diagnosis with a relatively poor prognosis owing to the absence of early clinical symptoms and effective screening methods. Hepatitis B virus (HBV) is the leading cause of incident cases of HCC and deaths worldwide (33%), followed by alcohol (30%), hepatitis C virus (HCV) (21%) and other reasons (16%) [3, 4].

The previous standard first-line systemic treatments for HCC were only lenvatinib and sorafenib [5–10]. However, targeted agents only conferred limited survival benefits [11–13]. In addition, the efficacy of sorafenib in patients with HBV-related HCC was revealed to be inferior to that in patients without HBV infection [11, 13]. Recently, immunotherapy is changing treatment strategies for many malignant tumors, and increasing evidence suggests that patients with HCC may benefit from these new therapies [14, 15]. Single-agent immune checkpoint inhibitors (ICIs) represented by programmed cell death 1(PD-1) and programmed cell death ligand 1 (PD-L1) inhibitors have been recently evaluated in HCC patients, and the results of clinical trials were disappointed [16–18]. A combination of ICIs and vascular endothelial growth factor (VEGF) inhibitors might promote an immune permissive environment and enhance ICI response [19, 20]. Therefore, immune-based combinations have been more striking [21]. In 2020, the IMbrave-150 trial demonstrated for the first-time that atezolizumab combined with bevacizumab is superior to sorafenib in the treatment of unresectable HCC and obtained clinically meaningful improvement in overall survival (OS) and progression-free survival (PFS), leading to its global approval [22]. Similar result has also been found in ORIENT-32 trial [23]. COSMIC-312 study reported that atezolizumab plus cabozantinib achieved a lack of improvement in OS compared to sorafenib [24]. In 2022, European society of medical oncology (ESMO) congress which updated the latest progress of first-line treatment regimens for HCC published primary results from Leap-002 and SHR-1210-III-310 studies [25, 26]. Leap-002 as a multicenter phase III study did not meet pre-specified statistical significance for primary endpoints of OS and PFS between lenvatinib plus pembrolizumab and lenvatinib in advanced HCC. Correspondingly, SHR-1210-III-310 study showed positive findings, that the combination of camrelizumab and apatinib in patients with advanced liver cancer demonstrated significant clinical benefits in terms of OS and PFS at the common primary endpoint.

Although some studies have displayed that combination therapy achieved better survival benefits than alone [27, 28]. Leap-002 and SHR-1210-III-310 study, global multicenter phase III clinical trials reported in ESMO 2022 have not been included in the previous research. Considering a large number of immunotherapy studies and new combination therapies for HCC exhibiting different clinical outcomes, we conducted this systematic review and meta-analysis aimed to overcome the limitations of individual research to better estimate the efficacy and safety of ICIs-combined anti-angiogenic therapy in treatment of unresectable HCC. Simultaneously, whether subgroups provided better OS and PFS outcomes were also explored to screen advantageous populations and determine the best therapy regimen.

## Materials and methods

This systematic review and meta-analysis were in accordance with the Preferred Reporting Items for Systematic Reviews and Meta-Analyses (PRISMA). This study was registered with the International Prospective Register of Systematic Reviews (PROSPERO CRD42023400568).

### Data source and search strategy

The databases, including Cochrane Library, PubMed, Embase and Web of Science were searched for eligible studies. The search time was from inception to November 2022. The main search therapy-related retrieval fields included (anti-angiogenic OR molecular targeted therapy OR targeted therapy) AND (PD-1 inhibitors OR programmed death ligand 1 OR PD-L1 inhibitors OR programmed death 1 receptor OR immunotherapy OR immune checkpoint inhibitors). The disease-related retrieval fields included hepatocellular carcinoma OR liver cell carcinoma OR Liver cancer. In addition, the reference lists of all relevant articles as well as conference abstracts published in main international oncological meetings (such as American Society of Clinical Oncology (ASCO), ASCO gastrointestinal cancer Symposium (ASCO-GI) and ESMO) were also searched to identify additional relevant studies.

### Study selection

Potential trials, with the exception of reviews (including meta-analysis), editorials, fundamental studies, animal studies, comments and case reports, were eligible to be included in this meta-analysis if all of the following criteria apply: (1) prospective phase III randomized controlled trials (RCTs); (2) diagnosis of unresectable HCC; (3) comparison with PD-1/PD-L1 inhibitors plus anti-angiogenic drugs and anti-angiogenic therapy alone; (4) clinical outcomes of the study including OS, PFS, objective response rate (ORR), disease control rate (DCR) and treatment-related adverse events (TRAEs); (5) English as study language.

### Data extraction and quality assessment

The following contents were extracted for each eligible study: (1) study general information (study name, first author, publication year, trial phase, study design, sample size); (2) basic information about the patients (age, male, etiology, geographical region); (3) interventions and control group. The main outcomes are PFS, OS, ORR, DCR and TRAEs. Both Response Evaluation Criteria in Solid Tumors version 1.1 (RECIST 1.1) and hepatocellular carcinoma-specific modified RECIST (mRECIST) criteria were used in the study. The risk of bias to verify methodological quality was evaluated based on the Cochrane Collaboration’s tool for randomized control trials by the Review Manager 5.4 (RevMan5.4).

### Statistical analysis

The statistical analysis was conducted using Stata 14.0 and RevMan5.4. The pooled hazard ratios (HRs) and 95% confidence interval (CI) for OS and PFS were calculated, as well as the pooled odds ratios (ORs) and 95% CI for ORR, DCR, any grade TRAEs and grade 3–5 TRAEs. Heterogeneity among studies was quantified by the *I*^*2*^test, and *I*^*2*^ > 50% and *p* < 0.05 was considered statistically significant heterogeneity [29]. When heterogeneity was significant, a random-effects model was used to calculate the pooled HR and OR; otherwise, the fixed-effects model was adopted.

Through subgroup analysis, publication bias assessment and sensitivity analysis, the origin of the heterogeneity was further explored. Begg’s test and Egger’s test were conducted to evaluate publication bias. The publication bias was absent with* p* > 0.05 in Begg’s test and Egger’s test [30]. We performed a sensitivity analysis by removing each study to observe changes in pooled HR. Region (Asia vs non-Asia), macrovascular invasion (MVI) or extra hepatic spread (EHS) (presence vs absence), alphafetoprotein (AFP) level (< 400 vs ≥ 400 ng per milliliter), etiology of HCC (HBV vs HCV vs Non-viral) and Barcelona Clinic Liver Cancer (BCLC) stage (B vs C) were considered in subgroup analysis.

## Results

### Study selection and characteristics

**Fig. 1:**
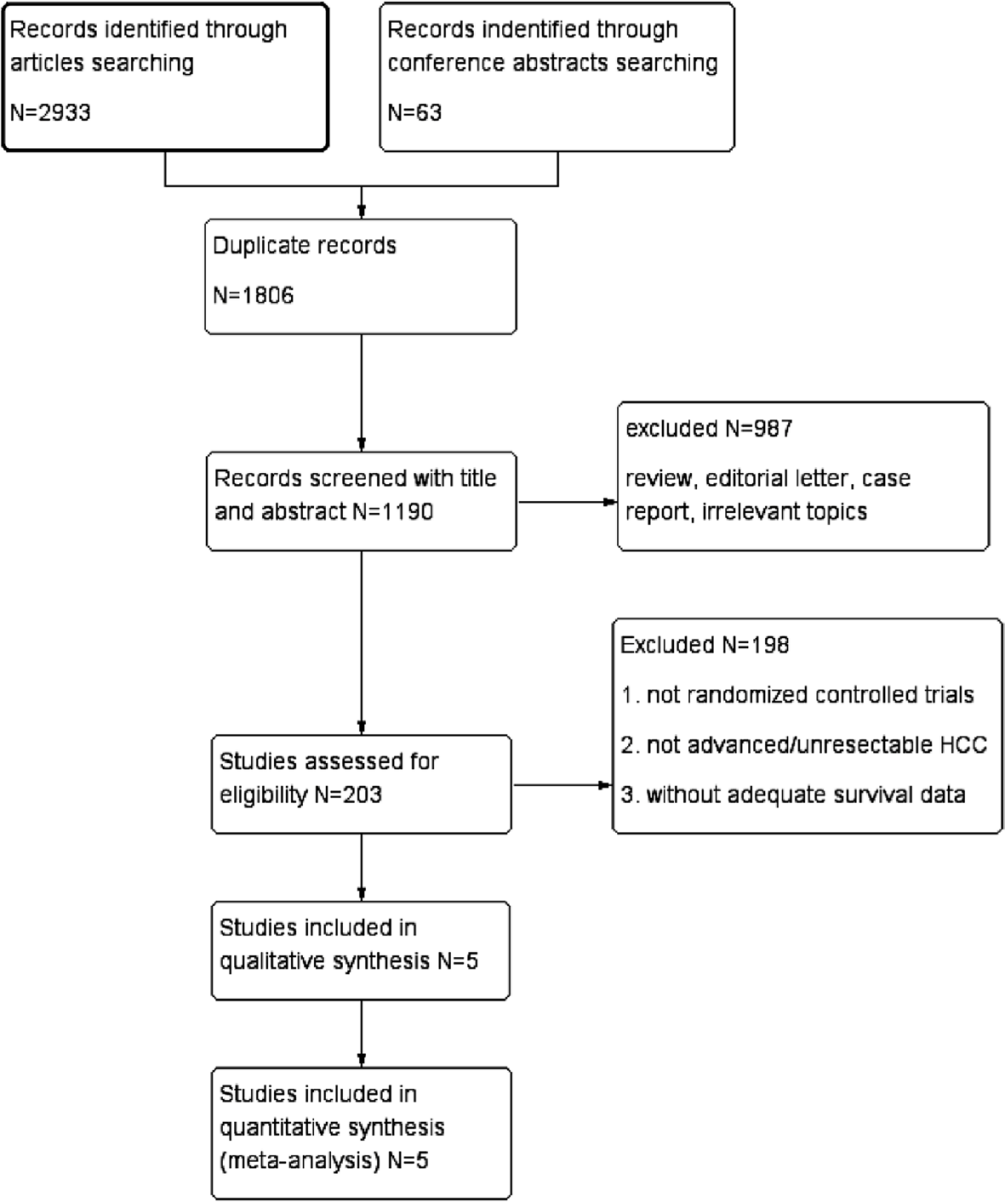
Flow diagram of the screening and selection process

A total of 2996 potential relevant reports were collected through two authors’ independent evaluation. After excluding duplicate and irrelevant studies, the initial search identified 1190 articles and abstracts. Finally, five studies were included among the 203 eligible full-text articles and conference abstracts (Fig. 1) [22–26].

The studies’ general information, baseline characteristics of patients and therapeutic regimen were recorded in Table 1. All studies were prospective phase III RCTs, with two reported in conference abstracts and three in articles. The studies were published between 2020 and 2022. There were up to 3057 patients available for the meta-analysis with a mean age around 61 years old.

**Table 1:** Main characteristic of the eligible studies in the meta-analysis

Study name/ Author	Year	Study Phase/design	Numbers of parents	Male (%)	Median age	Arm	Median OS	HR (95%CI)	Median PFS	HR (95%CI)	HBV + (%)	Asia (%)	Type of study
IMbrave150 Finn et al. [22]	2020	III/RCT	336	277(82%)	64	Atezolizumab + Bevacizumab	-	0.58 (0.42–0.79)	6.8	0.59 (0.47–0.76)	164(49%)	133 (40%)	Full text
			165	137(83%)	66	Sorafenib	13.2		4.3		76(46%)	68 (41%)	
ORIENT-32 Ren et al. [23]	2021	III/RCT	380	334(88%)	53	Sintilimab + Bevacizumab	-	0.57 (0.43–0.75)	4.6	0.56 (0.46–0.70)	359(94%)	380 (100%)	Full text
			191	171(90%)	54	Sorafenib	10.4		2.8		179(94%)	191 (100%)	
COSMIC-312 Kelley et al. [24]	2022	III/RCT	432	360(83)	64	Atezolizumab + Cabozantinib	15.4	0.90 (0.69–1.18)	6.8	0.63 (0.44–0.91)	127(29%)	120(28%)	Full text
			217	186(86)	64	Sorafenib	15.5		4.2		64(29%)	63(29%)	
Leap-002 Finn et al. [25]	2022	III/RCT	395	317(80.3%)	66	Pembrolizumab + Lenvatinib	21.2	0.840(0.71–1.0)	8.2	0.834 (0.71–0.98)	192(48.6%)	121(30.6%)	Absctrct
			399	327(82%)	66	Lenvatinib	19.0		8.1		193(48.4%)	123(30.8%)	
SHR-1210-III-310 Qin et al. [26]	2022	III/RCT	271	227(83.5%)	58	Camrelizumab + Apatinib	22.1	0.62 (0.49–0.80)	5.6	0.52 (0.41–0.65)	208(76.8%)	225(83.0%)	Absctrct
			271	230(84.9%)	56	Sorafenib	15.2		3.7		197(72.7%)	224(82.7%)	

### Risk of bias

Four studies were judged as having high risk for blinding participants and personnel blinding bias. One study was rated as unclear risk for the blinding of outcome assessment. The others were rated as low risk (Fig. S1).

### Meta-analysis of OS and PFS

**Fig. 2:**
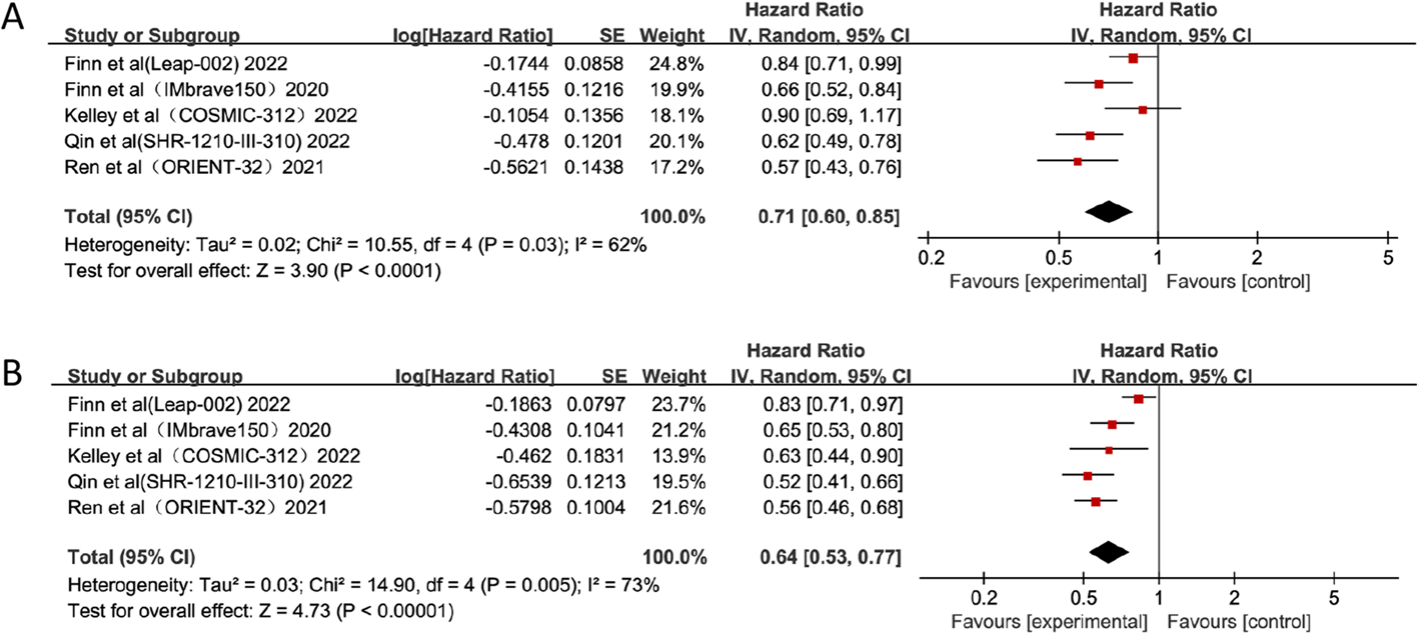
Forest plots of OS (**A**) and PFS (**B**) of combination therapy with ICIs and anti-angiogenic drugs

For all five trials, the pooled effects of HR for OS and PFS were available. The results revealed that combination therapy with PD-1/PD-L1 inhibitors and anti-angiogenic drugs had significantly better pooled OS than anti-angiogenic monotherapy (HR = 0.71; 95% CI: 0.60–0.85, *p* < 0.0001) (Fig. 2). Compared with anti-angiogenic monotherapy, combination therapy resulted in a significant improvement in PFS (HR = 0.64; 95% CI: 0.53–0.77, *p* < 0.00001) (Fig. 2). In addition, OS and PFS results showed a high degree of heterogeneities among the included studies (*I*^*2*^ = 62% and 73%, respectively). We performed subgroup analyses to determine the origin of heterogeneities among different studies.

### Meta-analysis of ORR and DCR

**Fig. 3:**
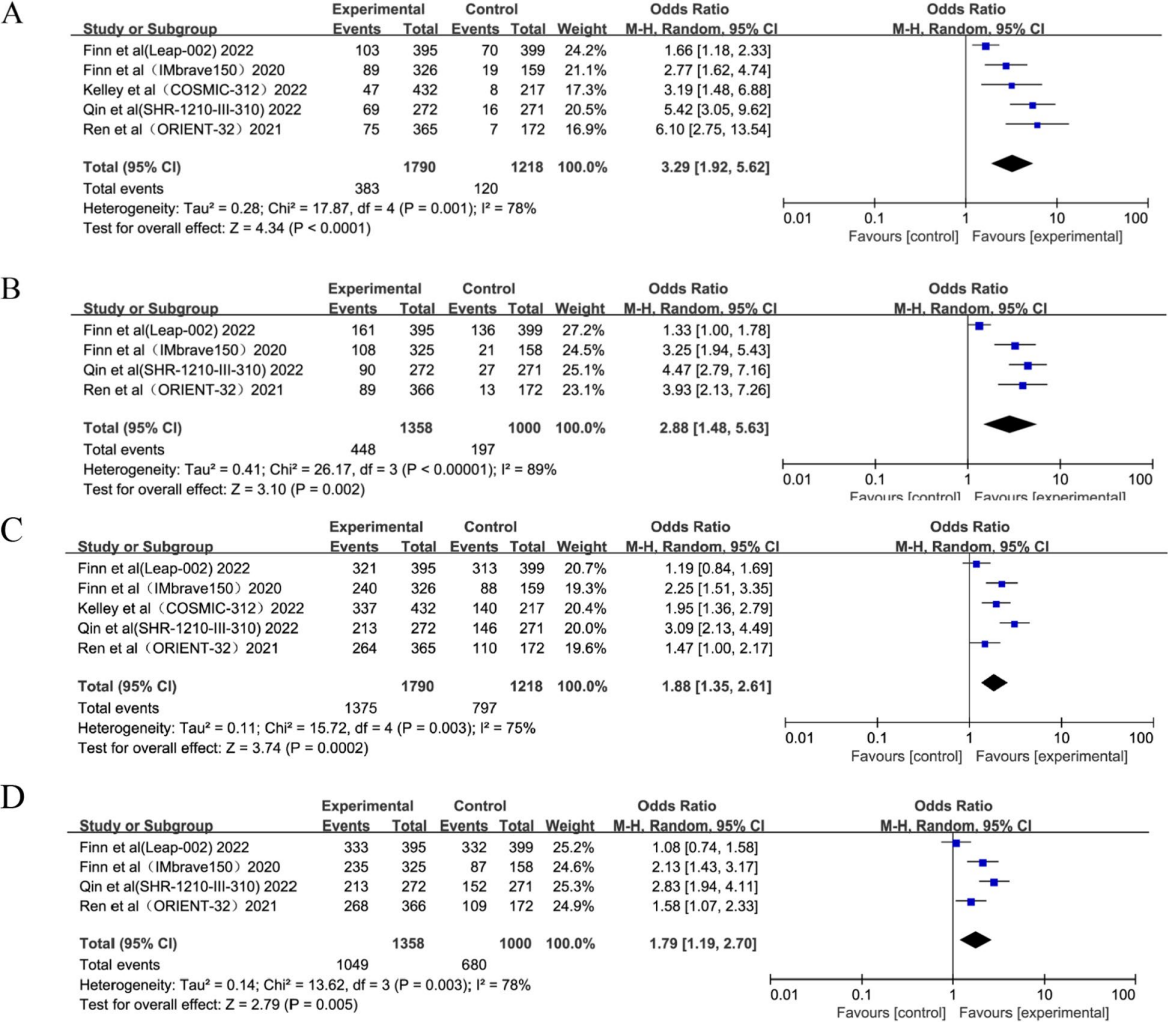
Forest plots of ORR by RECIST 1.1 (**A**), ORR by mRECIST (**B**), DCR by RECIST 1.1 (**C**) and DCR by mRECIST (**D**)

Four studies used both RECIST 1.1 and mRECIST methodologies to assess ORR, and one study used RECIST 1.1 alone. Interestingly, the combination therapy group generated respectable ORRs ((OR = 3.29; 95%CI: 1.92–5.62, *p* < 0.0001) and (OR = 2.88; 95%CI: 1.48–5.63, *p* = 0.002)) according to RECIST 1.1 and mRECIST, respectively (Fig. 3). The DCR was also assessed similarly to ORR. The pooled analysis revealed higher DCRs ((OR = 1.88; 95%CI: 1.35–2.61, *p* = 0.0002) and (OR = 1.79; 95%CI: 1.19–2.70, *p* = 0.005)) according to RECIST 1.1 and mRECIST in the combination therapy compared with anti-angiogenic monotherapy (Fig. 3).

### Subgroup analysis for OS

Subgroup analyses were performed for OS based on stratification factors (geographical region, presence of MVI or EHS, AFP level, etiology and BCLC Stage). The results were exhibited in Table 2 and Fig. S2. In MVI or EHS, AFP level and BCLC Stage subgroups, significant benefits of OS were observed in patients with combination treatment, while no significant reduction of heterogeneity. In terms of the HBV subgroup, the combination therapy displayed more benefits of OS (HR = 0.64; 95% CI: 0.55–0.74, *p* < 0.00001), however, there was no statistically significant difference between the combination treatment group and anti-angiogenic group in HCV (HR = 0.81; 95% CI: 0.64–1.04, *p* = 0.1) and non-viral subgroups (HR = 0.91; 95% CI: 0.75–1.11, *p* = 0.37). Meanwhile, the heterogeneities of the region subgroup and etiology subgroup were significantly reduced through subgroup analysis. A better OS benefit was demonstrated in Asian population and hepatitis B-positive population.

**Table 2:** The subgroup analysis for OS in patients with HCC

Subgroup		Number of studies	Pooled OS		Heterogeneity	
			HR[95% CI]	*p*	*I*^*2*^	*p*
Region	Asia	5	0.65[0.56, 0.76]	< 0.00001	0%	0.53
	Non-Asia	4	0.85[0.73, 0.98]	0.02	43%	0.15
AFP Lever	AFP < 400 ng/ml	4	0.75[0.65, 0.88]	0.0003	72%	0.01
	AFP ≥ 400 ng/ml	4	0.64[0.54, 0.77]	< 0.00001	0%	0.95
MVI or EHS	presence	5	0.67[0.59, 0.77]	< 0.00001	37%	0.17
	absence	5	0.78[0.65, 0.92]	0.003	68%	0.01
BCLC Stage	BCLC B	4	0.64[0.52, 0.79]	< 0.00001	45%	0.14
	BCLC C	4	0.70[0.61, 0.79]	< 0.00001	63%	0.04
Etiology	Hepatitis B	5	0.64[0.55, 0.74]	< 0.00001	0%	0.49
	Hepatitis C	4	0.81[0.64, 1.04]	0.1	58%	0.07
	Non-viral	4	0.91[0.75, 1.11]	0.37	0%	0.52

### Subgroup analysis for PFS

The results of the subgroup analysis for PFS were depicted in Table 3 and Fig. S3. The geographical region, presence of MVI or EHS, AFP level, etiology and BCLC Stage as subgroups revealed that patients treated with combination therapy showed better PFS than anti-angiogenic therapy. Only the non-viral subgroup showed no significant difference between the combination treatment group and the anti-angiogenic group (HR = 0.77, 95% CI: 0.59–1.0, *p* = 0.05). The heterogeneity of each subgroup was drastically reduced through subgroup analysis, especially the geographical region subgroup and etiology subgroup (*I*^*2*^ = 0%). This also suggested that hepatitis B-positive people have significantly more PFS benefit.

**Table 3:** The subgroup analysis for PFS in patients with HCC

Subgroup		Number of studies	Pooled PFS		Heterogeneity	
			HR[95% CI]	*p*	*I*^*2*^	*p*
Region	Asia	4	0.54[0.48, 0.62]	< 0.00001	0%	0.85
	Non-Asia	3	0.70[0.57, 0.86]	0.0006	0%	0.74
AFP Lever	AFP < 400 ng/ml	3	0.53[0.45, 0.63]	< 0.00001	60%	0.08
	AFP ≥ 400 ng/ml	3	0.61[0.50, 0.75]	< 0.00001	83%	0.003
MVI or EHS	presence	4	0.56[048, 0.66]	< 0.00001	0%	0.93
	absence	4	0.62[0.50, 0.75]	< 0.00001	41%	0.17
BCLC Stage	BCLC B	3	0.53[0.43, 0.67]	< 0.00001	34%	0.22
	BCLC C	3	0.59[0.49, 0.70]	< 0.00001	0%	0.80
Etiology	Hepatitis B	4	0.53[0.47, 0.59]	< 0.00001	0%	0.79
	Hepatitis C	3	0.65[0.45, 0.92]	0.02	0%	0.93
	Non-viral	3	0.77[0.59, 1.00]	0.05	0%	0.57

### Meta-analysis of TRAEs

**Fig. 4:**
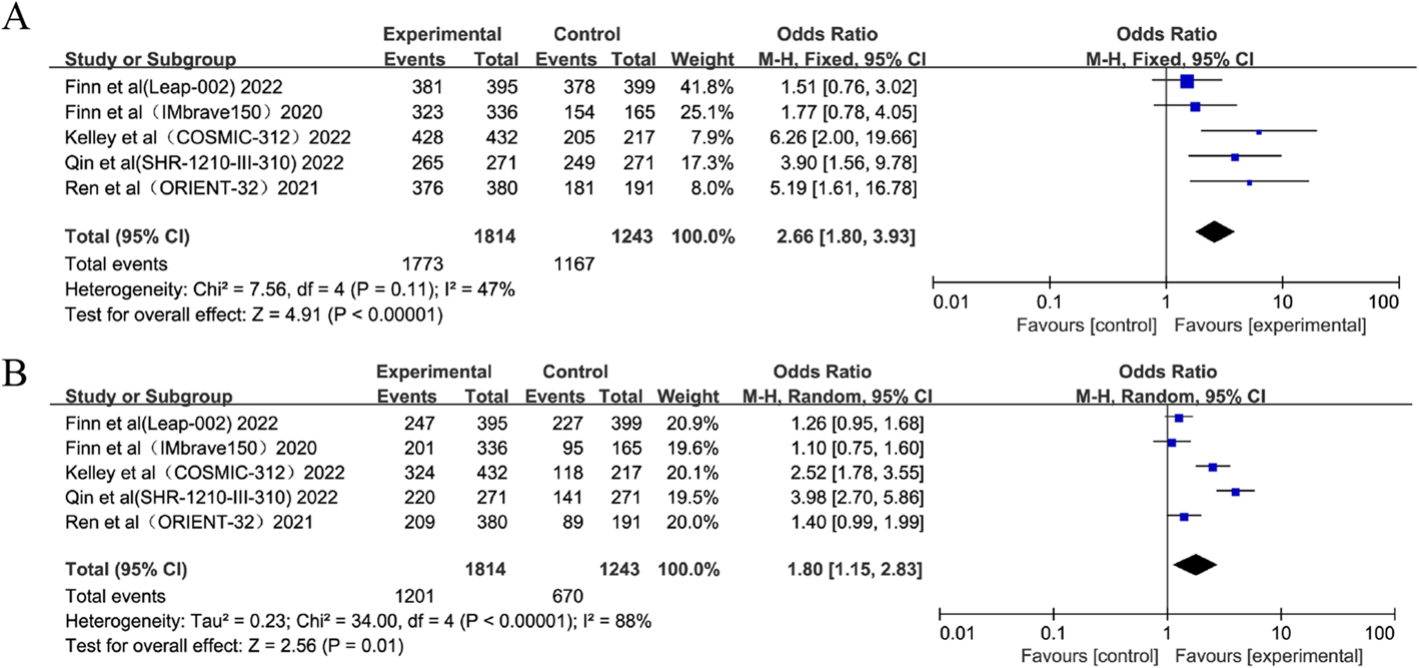
Forest plots of any grade (**A**) and grade 3–5 TRAEs (**B**)

All included trials recorded the incidences of any grade and grade 3–5 TRAEs. Combination therapy was associated with significantly higher incidences compared with anti-angiogenic monotherapy for both any grade TRAEs (OR = 2.66; 95% CI: 1.80–3.93, *p* < 0.00001) and grade 3–5 TRAEs (OR = 1.80; 95% CI: 1.15–2.83, *p* = 0.01) (Fig. 4).

### Sensitivity analysis and publication bias

Microvariation was observed in sensitivity analysis for the pooled effects by removing each trial in turn (Fig. S4). Publication biases were absent by Begg’s test (OS, *p* = 0.462; PFS, *p* = 0.462) and Egger’s test (OS, *p* = 0.315; PFS, *p* = 0.318).

## Discussion

Unresectable HCC accounts for approximately 75–85% of primary liver cancers, and treatment options are limited due to poor prognosis [31]. Finding appropriate treatment is necessary to improve patient survival [32]. Combination immunotherapy had a higher chance of being the most effective therapy than targeted monotherapy [22]. Recently, several ICI combination strategies for unresectable HCC have reported the encouraging results [22, 23, 26], but other results have been disappointing [24, 25]. Therefore, screening advantageous populations and determining the best combination therapy regimen has become a major challenge for HCC immunotherapy. We sought to find biomarkers or specific populations associated with immunoefficacy to stratify patients with HCC, distinguish between responders and non-responders, and recommend alternative therapies for patients who are not expected to respond to immunotherapy to avoid unnecessary toxicity. We conducted a meta-analysis which included five randomized controlled phase III trials of first-line therapies for unresectable HCC to show significantly better OS, PFS, ORR and DCR outcomes with PD-1/PD-L1 inhibitors in combination with anti-angiogenic drugs compared with anti-angiogenic drugs alone. Moreover, heterogeneities were revealed among the included studies for both OS and PFS. Subgroup analyses were performed to assess differences in outcomes and screen out the dominant population.

Theoretically, PD-L1 expression is the most direct marker for predicting the efficacy of PD-1/PD-L1 inhibitors, but unlike other malignancies, HCC is often accompanied by hepatitis or cirrhosis, which makes the tumor microenvironment of HCC more complex. The more complex classification of PD-L1 in HCC tissues and higher levels of spatial and cellular heterogeneity may affect the reliability and reproducibility of PD-L1 as a predictor of ICIs efficacy [33]. In the CheckMate 040 study [34], the ORR of PD-L1-positive patients was 26% in PD-L1-positive patients and 19% in negative patients, suggesting that negative expression of PD-L1 on tumor cells had no significant difference in the ORR against PD-1 therapy compared with PD-L1-positive patients. A phase II clinical trial of pembrolizumab in patients with unresectable advanced HCC suggested that there was no significant correlation between PD-L1 positivity and treatment response [35]. However, Zhou et al. reported a meta-analysis study showing that positive PD-L1 expression is better associated with ORR in patients with advanced liver cancer treated with anti-PD-1/PD-L1 [36]. Therefore, the expression of PD-L1 is currently controversial in predicting the efficacy of HCC. All included clinical trials in this meta-analysis lacked clinical outcomes in PD-L1-positive people. Therefore, PD-L1 expression was not included in the subgroup analysis in this study. Subgroup analyses were conducted according to the baseline characteristics of patients (geographical region, presence of MVI or EHS, AFP level, etiology and BCLC Stage).

In subgroup analysis, COSMIC-312 [24], IMbrave150 [22], SHR-1210-III-310 [26] and Leap-002 [25] were stratified for OS according to etiology (HBV, HCV and non-viral). The HRs of OS and PFS were stratified based on the factors of HBV status (positive vs negative) in the ORIENT-32 study [23]. Therefore, ORIENT-32 study was only included in the HBV subgroup. Subgroup analysis for PFS included COSMIC-312, IMbrave150 and SHR-1210-III-310. Leap-002 was not included due to the lack of data for each interested subgroup for PFS. It is found that combination therapy has no significant impact on the reduction of the risk of death compared with anti-angiogenic drugs in HCV patients. In the non-virus subgroup, there did not appear to be a difference between the combination therapy and anti-angiogenic treatment for OS and PFS. When considering only HBV-infected patients, combination therapies of all studies were confirmed to substantially reduce the risk of death compared to monotherapy, and the heterogeneity decreased substantially (*I*^*2*^ = 0%). Additionally, most patients in ORIENT-32 (94%) and SHR-1210-III-310 (76.8%) studies had HBV-related HCC, compared with less than 50% of participants in the Leap-002, IMbrave 150 and COSMIC-312 studies. This may be the reason for the better clinical outcomes of the ORIENT-32 and SHR-1210-III-310 studies. Basic studies have discovered that chronic HBV infection results in virus-specific T cell exhaustion and the PD-1/PD-L1 axis is a crucial inhibitor of HBV-specific CD8 + T cell activity [37]. Therefore, PD-1/PD-L1 inhibitors blocking could partially restore effective HBV-specific T-cell responses to viral proteins, which could theoretically affect the efficacy of ICIs [38, 39]. By contrast, non-viral HCC as a heterogeneous population that includes hepatic steatosis might be less responsive to immunotherapy compared with other etiologies of HCC [40]. HCV patients have wide geographical variations that exhibit different regional characteristics, such as metabolic syndrome and alcohol consumption, as well as anti-cancer treatments which might influence survival through both hepatic and extra hepatic effects or through follow-up therapy [41, 42]. Unlike previous meta-analyses that have not highlighted the characteristics of population, the present meta-analysis exclusively focused on differences in efficacy in subgroups of HBV, HCV and non-viral patients. Observations were extended to new combinations of therapeutic that were not covered in previous work.

COSMIC-312 [24], IMbrave150 [22], SHR-1210-III-310 [26] and Leap-002 [25] were included in subgroup analysis based on geographical region for the primary endpoints (OS). ORIENT-32 [23] study which was done for the Chinese population was only included in the Asian subgroup. Because of the lack of information for each subgroup of interest for PFS, Leap-002 was not included. The findings showed that combination therapy was significantly superior to monotherapy for OS in Asia, whereas there was no advantage benefit in patients with HCC of non-Asian population. For PFS, that combination therapy was significantly superior to monotherapy in both Asian and non-Asian population, however, pooled HR value was lower in Asia. In Africa and East Asia, the largest proportion of the population is attributable to be cause by HBV (60%); however, only 20% of cases in the Western world can be attributed to HBV infection, and chronic HCV is the most common potential liver disease etiology [4, 43]. Therefore, the perfect clinical outcomes of OS and PFS in the Asian population were also attributed to HBV infection being the dominant immunotherapy population. ORIENT-32 and SHR-1210-III-310 studies which had more patients in Asia (100% and 83.0%) differed from those in IMbrave150 (40%) study, COSMIC-312 (28%) study and Leap-002 (30.6%) study. This may induce the ORIENT-32 and SHR-1210-III-310 studies to have a lower HR value with positive outcomes.

Combination immunotherapy producing better clinical outcomes in patients with HBV-positive patients and Asian patients was discussed. However, there are still some patients who do not benefit from immunotherapy. Exosomes are closely related to viral hepatitis, cirrhosis and HCC. As an important intercellular communication mediator in the tumor immune microenvironment, exosomes may play a unique role in the immune response of HCC, thereby affecting the efficiency of immunotherapy. Exosomes exhibit the dual characteristics of tumor promotion and inhibition. On the one hand, they can mediate immunotherapy resistance by affecting the PD-1/PD-L1 axis or the anti-tumor function of immune cells in the tumor microenvironment. On the other hand, exosomes can carry drugs to downregulate PD-L1 expression on the surface of immune cells to improve the efficacy of ICI [44]. Unfortunately, however, there were no RCTs on exosome treatment under our search strategy. The relationship between exosome therapy and liver cancer (including HBV-related HCC) will be further explored in the future.

The mRECIST measured only the viable tumor, which is defined as the contrast-enhanced portion of the tumor on hepatic arterial phase images. However, RECIST 1.1 measured the whole lesion, which is not enough to evaluate therapy induced intratumoural necrosis [45, 46]. ESMO guidelines indicated the application of mRECIST or RECIST 1.1 in patients with HCC treated with anti-angiogenic targeted therapies [47]. However, National Comprehensive Cancer Network (NCCN) guidelines suggested that mRECIST and RECIST 1.1 are needed to assess tumor response of molecular targeted drugs [48]. The efficacies (ORR and DCR) of combination therapy compared with anti-angiogenic monotherapy for HCC were assessed according to both RECIST 1.1 and mRECIST in this meta-analysis.

The analysis results of TRAEs showed that compared with anti-angiogenic therapy, the combination therapy appears to have a significantly higher incidence of TRAEs. For these five trials, the most common TRAEs from combination therapies were hypertension, increased alanine aminotransferase, increased aspartate aminotransferase, proteinuria, diarrhea, fatigue, etc. Most of the TRAEs were concentrated in grade 1–2 indicating that the adverse events could be manageable. Grade 3–5 TRAEs occurred more frequently with camrelizumab plus apatinib in the SHR-1210-III-310 study (OR = 3.98; 95% CI: 2.70–5.86). The most common serious TRAEs were hypertension, increased alanine aminotransferase and increased aspartate aminotransferase.

There may be some possible limitations in this meta-analysis. Firstly, the RCTs selected in this meta-analysis involved various types of therapeutic drugs and diverse baseline characteristics, which may cause significant heterogeneities in data analysis in the aspect of the dissimilar clinical therapeutic effects and TRAEs. Therefore, the subgroup analyses were conducted attempting to stratify by baseline characteristics to mitigate the impact of heterogeneities. The efficacy and safety of the combination therapy can be further investigated through network meta-analysis in the future. Secondly, the present study included only five RCTs to compare PD-1/PD-L1 inhibitors combination therapy with anti-angiogenic monotherapy in patients with unresectable HCC. Further clinical trials would provide more reliable data for analysis, which may be included in future studies. Thirdly, there were inadequate cost-effective analyses for HCC in these trials, which might prove to be important for individual therapy. More cost-effective analyses are warranted due to the higher cost of the combination therapy than anti-angiogenic monotherapy. Lastly, there are inadequate mechanism reports of HBV response and resistance of immunotherapy. Future research will be critical for demonstrating the relationship between HBV infection and efficacy of ICIs.

## Conclusions

PD-1/PD-L1 inhibitors combination therapy for unresectable HCC was associated with better OS, PFS, ORR, and DCR than anti-angiogenic monotherapy, especially for the first-time discovery of better survival benefits for HBV infection and Asian population. Responses achieved with combination therapy may not have been more clinically meaningful to HCV infection and non-viral patients with unresectable HCC. The incidences of any grade and grade 3–5 TRAEs were significantly higher in patients receiving combination therapy, but the safety was manageable. This meta-analysis provides new treatment options for unresectable HCC patients, especially for those with HBV-associated HCC.

## Supplementary Information

**Additional file 1: Figure S1. **Risk of bias summary (A) and risk of bias graph (B). **Figure S2.** Forest plots of HRs comparison of OS between PD-1/PD-L1 inhibitors plus anti-angiogenic group and anti-angiogenic group in subgroup analysis (A) region, (B) MVI or EHS, (C) AFP Level, (D) Etiology, (E) BCLC stage. **Figure S3.** Forest plots of HRs comparison of PFS between PD-1/PD-L1 inhibitors plus anti-angiogenic group and anti-angiogenic group in subgroup analysis (A) region, (B) MVI or EHS, (C) AFP Level, (D) Etiology, (E) BCLC stage. **Figure S4.** Pooled HRs of OS (A) and PFS (B) in sensitivity analysis.

## Data Availability

The original datasets for this study are included in the article/Supplementary Material.

## Abbreviations

HCC: Hepatocellular carcinoma
HBV: Hepatitis B virus
ICI: Immune checkpoint inhibitor
HR: Hazard ratio
CI: Confidence intervals
OS: Overall survival
PFS: Progression-free survival
OR: Odds ratio
ORR: Objective response rate
DCR: Disease control rate
TRAEs: Treatment-related adverse events
HCV: Hepatitis C virus
VEGF: Vascular endothelial growth factor
PD-1: Programmed cell death 1
PD-L1: Programmed cell death ligand 1
ASCO: American Society of Clinical Oncology
ESMO: European society of medical oncology
ASCO-GI: American Society of Clinical Oncology gastrointestinal cancer Symposium
RCTs: Random control trials
RECIST 1.1: Response Evaluation Criteria in Solid Tumors version 1.1
mRECIST: Hepatocellular carcinoma-specific modified RECIST
MVI: Macrovascular invasion
EHS: Extra hepatic spread
AFP: Alphafetoprotein
BCLC: Barcelona Clinic Liver Cancer
NCCN: National Comprehensive Cancer Network
