# Supplementary material for: Efficacy and safety of PD-1/PD-L1 inhibitors combined with anti-angiogenic therapy for the unresectable hepatocellular carcinoma and the benefit for hepatitis B virus etiology subgroup: a systematic review and meta-analysis of randomized controlled trials
Source: BMC Cancer. 2023 May 24;23:474. doi: 10.1186/s12885-023-10960-w (PMC10207853; doi:10.1186/s12885-023-10960-w)
Supplement: Supplementary file 1 — Additional file 1: Figure S1. Risk of bias summary (A) and risk of bias graph (B). Figure S2. Forest plots of HRs comparison of OS between PD-1/PD-L1 inhibitors plus anti-angiogenic group and anti-angiogenic group in subgroup analysis (A) region, (B) MVI or EHS, (C) AFP Level, (D) Etiology, (E) BCLC stage. Figure S3. Forest plots of HRs comparison of PFS between PD-1/PD-L1 inhibitors plus anti-angiogenic group and anti-angiogenic group in subgroup analysis (A) region, (B) MVI or EHS, (C) AFP Level, (D) Etiology, (E) BCLC stage. Figure S4. Pooled HRs of OS (A) and PFS (B) in sensitivity analysis. [file 12885_2023_10960_MOESM1_ESM.docx]

**Supplementary Information**

**
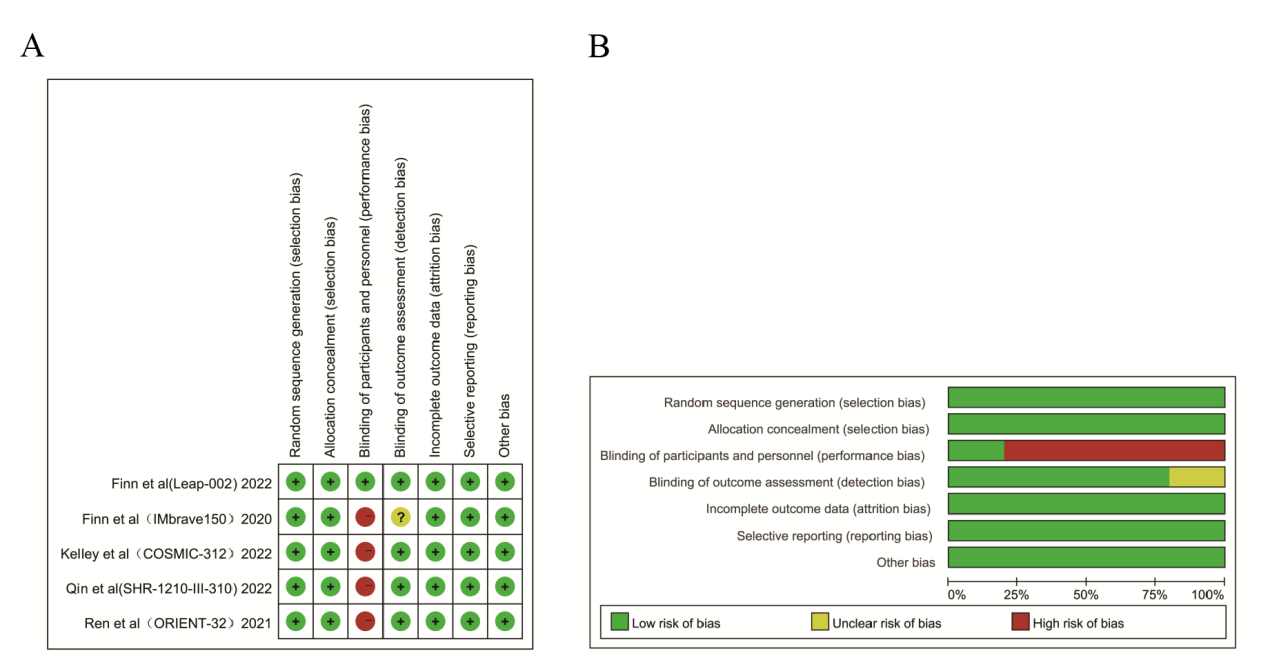
**

**Figure S1** Risk of bias summary (A) and risk of bias graph (B)

**
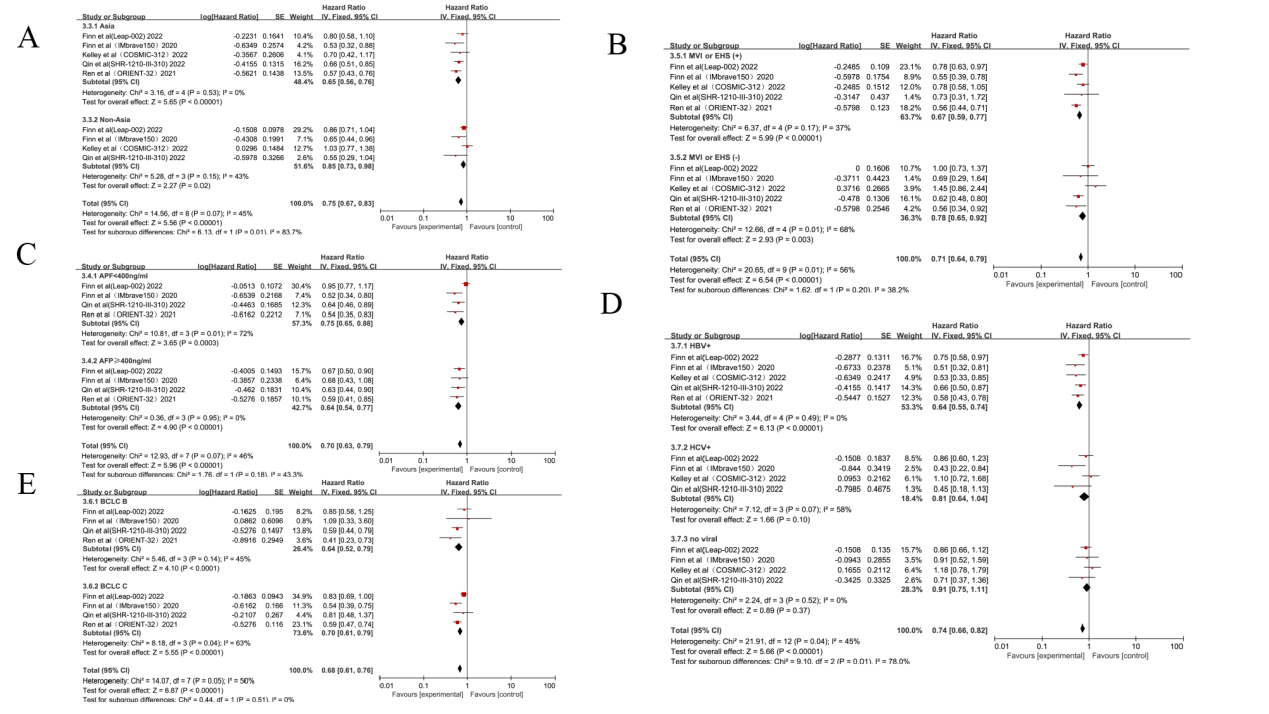
**

**Figure S2** Forest plots of HRs comparison of OS between PD-1/PD-L1 inhibitors plus anti-angiogenic group and anti-angiogenic group in subgroup analysis (A) region, (B) MVI or EHS, (C) AFP Level, (D) Etiology, (E) BCLC stage


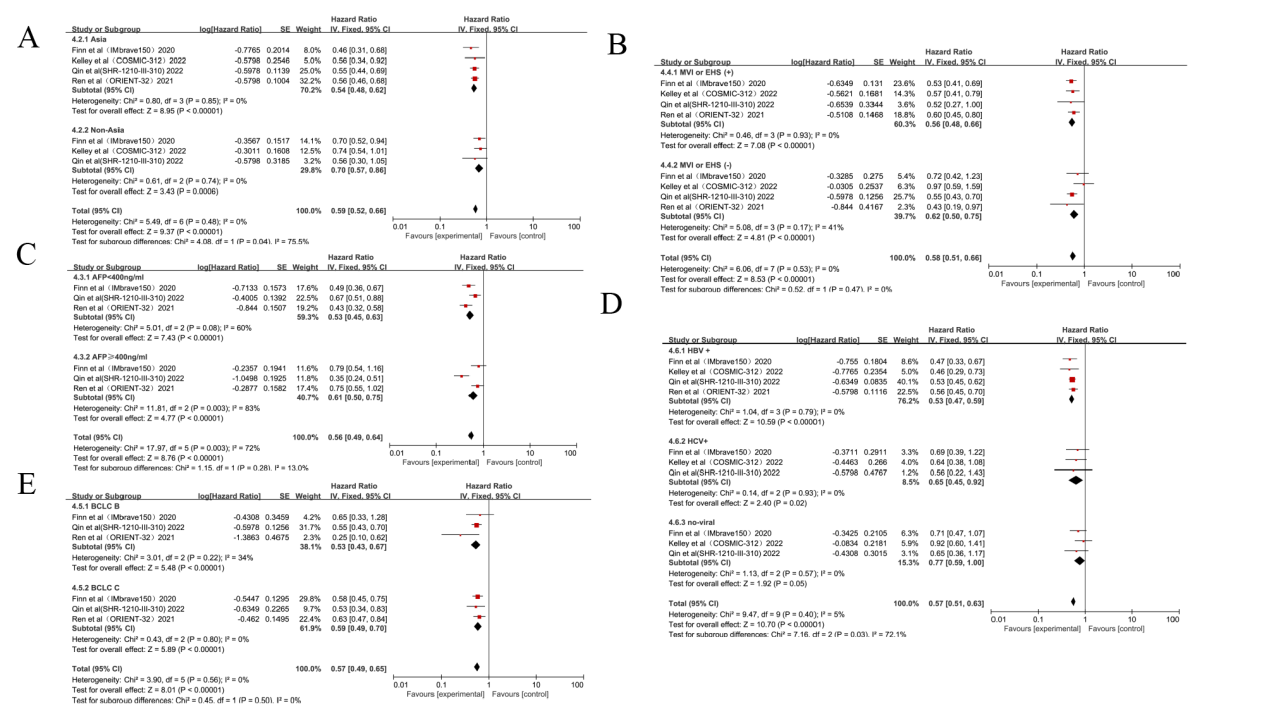


**Figure S3** Forest plots of HRs comparison of PFS between PD-1/PD-L1 inhibitors plus anti-angiogenic group and anti-angiogenic group in subgroup analysis (A) region, (B) MVI or EHS, (C) AFP Level, (D) Etiology, (E) BCLC stage


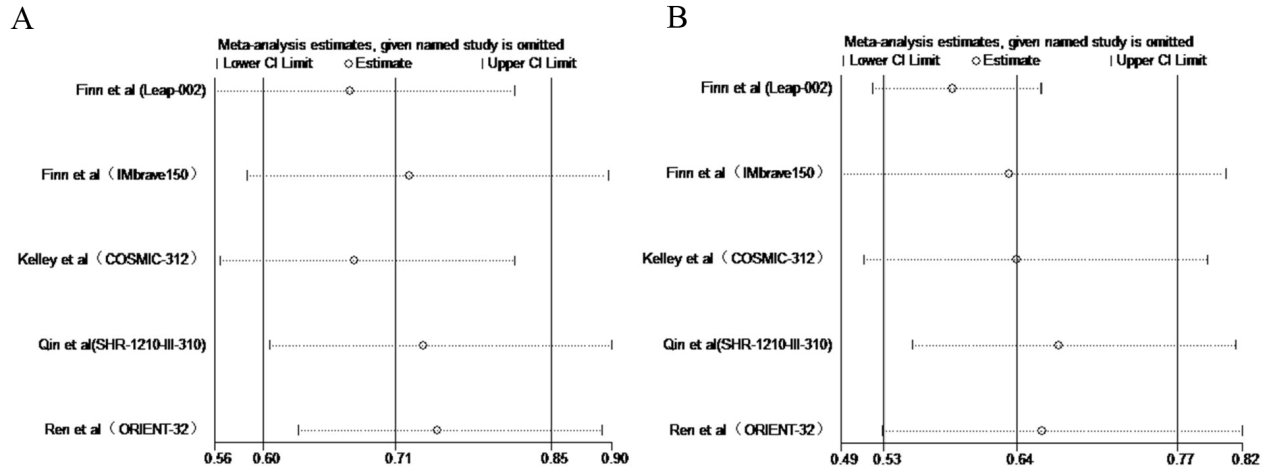


**Figure S4** Pooled HRs of OS (A) and PFS (B) in sensitivity analysis
